# Club Cell Protein 16 Attenuates CD16^bright^CD62^dim^ Immunosuppressive Neutrophils in Damaged Tissue upon Posttraumatic Sepsis-Induced Lung Injury

**DOI:** 10.1155/2021/6647753

**Published:** 2021-01-28

**Authors:** Nils Becker, Philipp Störmann, Andrea Janicova, Kernt Köhler, Klemens Horst, Ildiko Rita Dunay, Claudia Neunaber, Ingo Marzi, Jan Tilmann Vollrath, Borna Relja

**Affiliations:** ^1^Experimental Radiology, Department of Radiology and Nuclear Medicine, Otto von Guericke University, Magdeburg, Germany; ^2^Department of Trauma, Hand and Reconstructive Surgery, University Hospital Frankfurt, Germany; ^3^Institute of Veterinary Pathology, Justus Liebig University Giessen, Giessen, Germany; ^4^Department of Trauma Surgery, Hospital of the RWTH University, Aachen, Germany; ^5^Institute of Inflammation and Neurodegeneration, Otto von Guericke University, Magdeburg, Germany; ^6^Trauma Department, Hannover Medical School, Hannover, Germany

## Abstract

**Background:**

Recently, identification of immunosuppressive polymorphonuclear leukocytes (PMNL) that were traditionally described as proinflammatory cells emerged in the field of posttraumatic immunity. To understand their local and remote distribution after trauma, PMNL-subsets and the impact of immunomodulatory Club Cell protein (CC)16 that correlates with pulmonary complications were assessed.

**Methods:**

C57BL/6N mice were divided into three groups, receiving isolated blunt chest trauma (TxT), undergoing TxT followed by cecal ligation and puncture (CLP, TxT + CLP) after 24 h, or sham undergoing analgosedation (*n* = 18/group). Further, each group was subdivided into three groups receiving either no treatment (ctrl) or intratracheal neutralization of CC16 by application of anti-CC16-antibody or application of an unspecific IgG control antibody (*n* = 6/group). Treatment was set at the time point after TxT. Analyses followed 6 h post-CLP. PMNL were characterized via expression of CD11b, CD16, CD45, CD62L, and Ly6G by flow cytometry in bone marrow (BM), blood, spleen, lung, liver, and bronchoalveolar and peritoneal lavage fluid (BALF and PL). Apoptosis was assessed by activated (cleaved) caspase-3. Results from untreated ctrl and IgG-treated mice were statistically comparable between all corresponding sham, TxT, and TxT + CLP groups.

**Results:**

Immature (CD16^dim^CD62L^bright^) PMNL increased significantly in BM, circulation, and spleen after TxT *vs*. sham and were significantly attenuated in the lungs, BALF, PL, and liver. Classical-shaped (CD16^bright^CD62L^bright^) PMNL increased after TxT *vs*. sham in peripheral tissue and were significantly attenuated in circulation, proposing a trauma-induced migration of mature or peripheral differentiation of circulating immature PMNL. Immunosuppressive (CD16^bright^CD62L^dim^) PMNL decreased significantly in the lungs and spleen, while they systemically increased after TxT *vs*. sham. CLP in the TxT + CLP group reduced immunosuppressive PMNL in PL and increased their circulatory rate *vs*. isolated TxT, showing local reduction in affected tissue and their increase in nonaffected tissue. CC16 neutralization enhanced the fraction of immunosuppressive PMNL following TxT *vs*. sham and decreased caspase-3 in the lungs post-CLP in the TxT + CLP group, while apoptotic cells in the liver diminished post-TxT. Posttraumatic CC16 neutralization promotes the subset of immunosuppressive PMNL and antagonizes their posttraumatic distribution.

**Conclusion:**

Since CC16 affects both the distribution of PMNL subsets and apoptosis in tissues after trauma, it may constitute as a novel target to beneficially shape the posttraumatic tissue microenvironment and homeostasis to improving outcomes.

## 1. Introduction

In 2016, more than 3 million people worldwide died directly after trauma or in consequence from its clinical complications [1]. The posttraumatic mortality has been illustrated by a biphasic distribution with direct or early and late in-hospital mortality [2]. As early mortality is mainly caused by injury severity, seriously injured trauma patients, surviving the initial phase, are at high risk for complications, e.g., multiple organ dysfunction syndrome, multiple organ failure (MOF), or sepsis caused by immunological dysregulations [2, 3]. Trauma-induced tissue damage provokes a massive release of endogenous damage-associated molecular patterns to initiate the resolution of nonpathogenic and pathogenic inflammation with subsequent tissue repair. Simultaneous abundance of a proinflammatory state and a compensatory, anti-inflammatory response syndrome results in a complex postinjury immune response. However, a systemic hyperinflammation may induce the remote organ damage and MOF [4, 5], while an excessive immunosuppression promotes the development of infectious complications and sepsis [6, 7]. Here, improved understanding of the regulatory mechanisms is crucial for the development of therapeutic strategies to prevent complications caused by the posttraumatic inflammatory dysregulation.

It is known that distinct protein levels correlate with specific damage patterns, e.g., systemic elevation of the Club Cell protein (CC)16 in patients suffering traumatic lung injury exemplifies [8]. CC16 is a 15.8 kDa protein secreted primarily by nonciliated club cells along the tracheobronchial epithelium, especially in distal respiratory and terminal bronchioles [9, 10]. It may serve as a biomarker to indicate the development of secondary pulmonary complications after trauma, but it also exerts anti-inflammatory and immunosuppressive properties [11–13]. Due to its anti-inflammatory effects, CC16 has been described to be protective in the development of chronic obstructive pulmonary disease in human and mouse [13, 14].

Latest findings suggest that myeloid-derived suppressive cells (MDSC) with immunosuppressive properties are increased in patients suffering from persistent inflammation, immunosuppression, and catabolism syndrome (PICS), which is associated with macrophage paralysis and recurrent infections [15–17]. The heterogeneous MDSC populations are divided into cells with characteristics of monocytes, Mo-MDSC, and so-called granulocyte- (G-) MDSC or polymorphonuclear- (PMN-) MDSC with granulocyte characteristics, respectively [18]. PMN-MDSC are defined as a subpopulation of PMNL [19], bringing a new diversity into the most frequent leukocyte fraction in human circulation, that has traditionally been considered a proinflammatory population of early effector cells [20]. However, the identification of immunosuppressive PMNL remains challenging, since different surface markers were shown to indicate immunosuppressive features. In mice, MDSC are described as Gr-1^+^CD11b^+^ [21, 22], with Ly6G^−^Ly6C^high^ defining Mo-MDSC and Ly6G^+^Ly6C^low^ defining PMN-MDSC [18]. As Ly6G is a neutrophil marker only expressed on murine PMNL, the identification of human MDSC and especially human PMNL with similar immunosuppressive functions is more complicated and still lacking an internationally approved unified surface recognition pattern [23]. A promising strategy to identify immunosuppressive PMNL in human was described by Pillay et al. in healthy volunteers who have been challenged with endotoxin, inducing a CD16^bright^CD62L^dim^-expressing population with a nuclear morphologic of hyper-segmented, mature PMNL. This cell population was shown to exhibit immune-suppressive properties as it reduced the T-cell proliferation [24]. CD16^dim^CD62L^bright^ cells had a banded nucleus morphology as described for immature PMNL [24], while CD16^bright^CD62L^bright^ exhibited a classical, segmented nuclear morphology, both without immunosuppressive properties. As PMNL are rapidly responding immune cells [25], their recently described functional heterogeneity is likely to affect the early immune regulation and priming of the long-term immune dysregulation after trauma.

We hypothesized that local trauma will change the dynamic distribution of PMNL subsets in affected and unaffected tissue in a murine trauma model of sepsis after blunt chest trauma. Due to its local and systemic immune-modulating characteristics, we also hypothesized the lung injury-associated CC16 to influence the PMNL subset distribution. Thus, a possible prevention of the posttraumatic immune dysregulation via CC16 may uncover a novel therapeutic approach to improve outcomes in the future.

## 2. Experimental Section

### 2.1. Animals and Ethics

54 male C57BL/6N mice (25 ± 5 g), 6-8 weeks of age, were provided by Janvier Labs (France) and held at the *Zentrale Forschungseinrichtung* at the University Hospital of the Goethe University Frankfurt, Germany. All animal experiments were approved by the Veterinary Department of the regional council *Regierungspräsidium Darmstadt* and were performed according to the German Federal Law and in consent with the ARRIVE guidelines [26]. The mice had access to water and food *ad libitum*. After experiments, mice received analgesia as described before [27].

### 2.2. Group Allocation and Experimental Model

The animals were randomly assigned into the isolated blunt chest/thoracic trauma (TxT) group (*n* = 18) with sterile laparotomy, the double-hit group consisting of TxT with subsequent cecal ligation and puncture (CLP, TxT + CLP) (*n* = 18) or the sham group undergoing analgosedation only (*n* = 18). All mice received analgesia with buprenorphin and a general mask anesthesia with isoflurane as described before [27, 28]. TxT and the double-hit groups of mice (TxT + CLP) received a bilateral blunt chest trauma as described before [28, 29]. Briefly, after supine positioning, 2.5 cm under a cylinder, a standardized blast wave was focused centrally to the chest. The air blast wave perforates the 0.05 mm Mylar polyester film and provides the air blast to the thorax (Du Pont, Bad Homburg, Germany).

**Figure 1:**
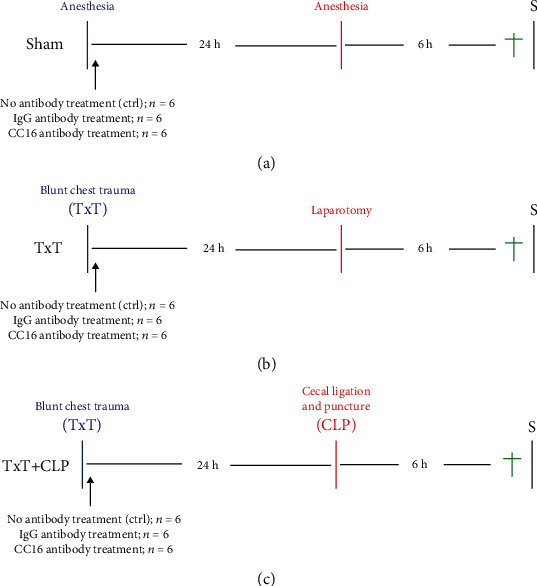
Experimental design. (a) In the sham group, eighteen animals underwent analgosedation, while 18 animals were randomly assigned into the isolated blunt chest/thoracic trauma (TxT) group (*n* = 18) with sterile laparotomy (b) and 18 animals into the double-hit group consisting of TxT with subsequent cecal ligation and puncture (TxT + CLP, c). In each of those groups, three further subgroups were created with *n* = 6 per group. One control subgroup received no treatment with antibodies (ctrl), the second subgroup received an unspecific IgG antibody, and the third group underwent intratracheal neutralization of CC16 by application of anti-CC16-antibody. The treatment was performed subsequently to TxT or corresponding to that timing in the sham subgroups.

Mice in the sham, TxT, or TxT + CLP groups underwent a randomization each into three further subgroups of *n* = 6 per group. One group of mice received no treatment (ctrl, *n* = 6), and the second group underwent intratracheal neutralization of CC16 by application of anti-CC16 antibody (*n* = 6), while a third subgroup received an unspecific IgG control antibody (*n* = 6). This treatment was performed subsequently to TxT or corresponding to that timing in the sham subgroups. Local application of 50 *μ*l Uteroglobin/SCGB1A1 (CC16 Ab, LS Biosciences) antibody in the intervention group or 50 *μ*l of the IgG (IgG) control antibody (10 *μ*g/ml, R&D Systems) was administered using an intratracheally placed buttoned cannula at the beginning of the trachea. Antibody distribution was ensured by positioning the mice in a supine position and keeping thoroughly the tongue aside. After a careful administration of the antibody, the mice were kept in the reverse Trendelenburg position for 30 seconds to assure the antibody distribution inside the lungs. 24 hours later, in general anesthesia (intraperitoneal application of ketamine, Zoetis, Berlin, Germany and xylazin, Bayer, Leverkusen, Germany), the double hit group (TxT + CLP) received additional CLP as described before [28, 30]. Briefly, after median laparotomy, the distal cecum underwent ligation and puncture with a 25-gauge cannula (Braun, Melsungen, Germany). The TxT group underwent sterile laparotomy using identical general anesthesia. Sham animals received only anesthesia. Six hours later all mice received intraperitoneal analgesic sedation as described above and euthanasia followed to facilitate sampling. For randomization of the animals, one animal per each group underwent the experimental procedure, and by this protocol, the group allocation was done. The experimental design is shown in Figure 1. For assessing relative changes in the PMNL distribution, sham animals receiving no antibodies were compared against the TxT or TxT + CLP groups with no antibody application. The results from those untreated controls and IgG-treated mice were statistically comparable between all corresponding sham, TxT, and TxT + CLP groups; therefore, in the following manuscript, the data are provided for the IgG-groups. Thus, to examine the effect of CC16 neutralization, animals receiving the IgG-control antibody were taken as the reference cohort and compared with animals receiving the anti-CC16 antibody.

### 2.3. Sampling

Intraperitoneal lavage (PL) was gained by puncturing the peritoneum with a 23-gauge cannula (Braun, Melsungen, Germany), flushing the abdominal cavity with 1.5 ml of phosphate-buffered saline (PBS). To collect the bronchoalveolar lavage fluid (BALF), the trachea was intubated using a 20-gauge indwelling venous cannula (Braun, Melsungen, Germany) and the lungs were flushed with 1.2 ml PBS. BALF and PL were centrifuged (1164 g at 4°C for 5 minutes). Cell pellets were resuspended in 100 *μ*l PBS supplemented with 0.5% bovine serum albumin (FACS buffer). 40 *μ*l was transferred into polystyrene FACS tubes (BD Pharmingen™) for further staining as described below.

Blood collection was performed by a *Vena cava* puncture with a 23-gauge cannula and heparinized syringe. Samples were centrifuged for 15 minutes at 1164 g and 4°C. The cellular pellet was resuspended in PBS (isovolume), and 30 *μ*l of whole blood (WB) was stored in FACS tubes for staining.

After blood sampling, the mice were perfused with 20 ml PBS *via* the cannula located in the *vena cava*. 25 mg of one lung lobe, one liver lobe, and spleen were taken and processed by Minute Single Cell Isolation protocol (Invent Biotechnologies, Minnesota, US). 40 *μ*l of isolated and in FACS buffer resuspended single cell pellets was stained for flow cytometric analysis as described below. For bone marrow (BM) sampling, the femoral bone was taken, and both diaphyses were removed. The bone cavity was subsequently flushed with 1 ml of PBS. To remove remaining bone material, the bone marrow was filtered using single cell isolation kit filter tubes (Invent Biotechnologies). The filtrate was centrifuged at 1164 g at 4°C for 5 minutes, and the pellet was resuspended in 100 *μ*l FACS buffer. 40 *μ*l was taken for further staining.

After blood withdrawal, one lung lobe was filled with 4% formalin for overnight fixation and immunohistological caspase-3 staining as described below. Since the left liver lobe was ligated and processed for FACS stainings, the remaining liver was flushed with 20 ml 10% buffered formalin solution and removed for further handling for immunohistology as described below.

### 2.4. FACS Staining

All samples were stored on ice and incubated with Alexa Fluor® 647-conjugated anti-mouse CD11b antibody (Ab) (BioLegend, San Diego, California, US), APC/Fire 750 conjugated anti-mouse CD45 Ab (BioLegend), Pacific Blue-conjugated anti-mouse Ly-6G/Ly6C Ab (Gr-1, RB6-8C5, BioLegend), Alexa Fluor® 488-conjugated anti-mouse CD62L Ab (Biolegend), and phycoerythrin- (PE-) conjugated anti-mouse CD16/CD32 Ab (BD Biosciences, Franklin Lakes, USA). After 30 minutes in the dark, 5 *μ*l 7-AAD (BD Biosciences) was added and samples were incubated for additional 15 minutes. 2 ml of FACS buffer was used for washing at room temperature (RT) with centrifugation at 423 g. After discarding of the supernatant 1 ml FACS Lysing Solution (BD Biosciences) was added to the cell pellet and incubated for 10 minutes at RT. The stained samples were centrifuged for 7 minutes at 400 g and washed twice with 2 ml FACS buffer. The remaining cell pellet was resuspended in 80 *μ*l FACS buffer and stored light-protected on ice until flow cytometric analysis.

### 2.5. FACS Analyzing and Gating Strategy

**Figure 2:**
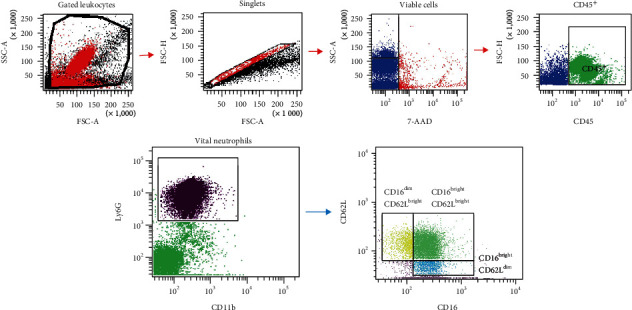
Gating strategy. Singlet leukocytes were identified by using forward and side scatter scan. Viability was detected by 7-AAD. Then, CD45^+^ cells (leukocytes) were gated by their Ly6G-expression to identify Ly6G^+^ population (PMNL fraction). Among the PMNL fraction, CD11b^+^ cells were divided by their relative expression of CD16 and CD62L, and distribution of CD16^dim^CD62L^bright^ (immature PMNL), CD16^bright^CD62L^bright^ (classical PMNL), and CD16^bright^CD62L^dim^ (immunosuppressive PMNL) cells among viable CD11b^+^PMNL was measured.

Stained single-cell isolations were analyzed using BD FACS Canto 2™ and BD FACS DIVA™ software. Cut-offs for fluorescence intensity were set by using the corresponding isotype control antibodies. In each sample, a minimum of 3.0 × 10^4^ cells were measured, despite few samples with lower total cellular amount. Singlets were identified by using a forward and side scatter scan. Viability was identified by isotype-controlled 7-AAD negativity. Next, CD45^+^ cells were gated by their Ly6G-expression to identify percentage of Ly6G^+^ cells (referred to as PMNL fraction). Among the PMNL fraction, CD11b^+^ cells were divided by their relative expression of CD16 and CD62L. In every sample, gating was performed by individual expression intensity according cell distribution as described by Pillay et al. [24] and as shown in Figure 2. Percental distribution of CD16^dim^CD62L^bright^ cells (immature PMNL), CD16^bright^CD62L^bright^ cells (classical PMNL), and CD16^bright^CD62L^dim^ cells (immunosuppressive PMNL), among CD11b^+^ PMNL, was measured.

### 2.6. Immunohistological Staining of Caspase-3

For the determination of the caspase-3 expression in the liver and lungs, paraffin-embedded sections were deparaffinized, rehydrated, and stained with antibodies as described below. Antigen retrieval was performed by R-universal solution (Aptum Biologics) and under steam atmosphere for 20 minutes (Retriever 2010). In order to block the endogenous peroxidase activity, hydrogen peroxide was applied (Peroxidase UltraVision Block). Anti-Cleaved Caspase-3 (Asp175) (#9661, Cell Signaling Technology) in Antibody Diluent with Background Reducing Components (Dako) were used as primary antibodies for 1 hour at room temperature. The secondary horseradish peroxidase-linked antibody (rabbit, Histofine Simple Stain, Nichirei Biosciences Inc.) was used to detect the cleaved caspase-3. 3-Amino-9-ethylcarbazol (AEC, DCS Innovative Diagnostik-Systeme, Hamburg) was applied as a substrate to detect specific binding. The samples were finally counterstained with hematoxylin. The mean number of positively stained cells/high power field was assessed from 25 different fields per slide.

### 2.7. Statistical Analysis

Statistical analysis was performed using GraphPad Prism 6 (GraphPad Software, Inc., San Diego, CA). Based on the histogram and Shapiro-Wilk test, the nonparametric Kruskal-Wallis test, not assuming a normal distribution of the residuals, followed by Dunn's post hoc test for the correction of multiple comparisons was applied. Results are given as the mean and standard error of the mean (SEM). A *p* value below 0.05 was considered statistically significant.

## 3. Results

### 3.1. Main Findings

For assessing relative changes in the PMNL distribution, sham animals receiving no antibodies were compared against the TxT or TxT + CLP groups with no antibody application. The results from those untreated controls and IgG-treated mice were statistically comparable between all the corresponding sham, TxT, and TxT + CLP groups (data not shown); therefore, in the following manuscript, the data are provided for the IgG-groups. Thus, to examine the effect of CC16 neutralization, animals receiving the IgG-control antibody were taken as the reference cohort and compared with animals receiving the anti-CC16 antibody.

#### 3.1.1. Trauma Induced Systemic Recruitment of PMNL Out of the Bone Marrow

**Figure 3:**
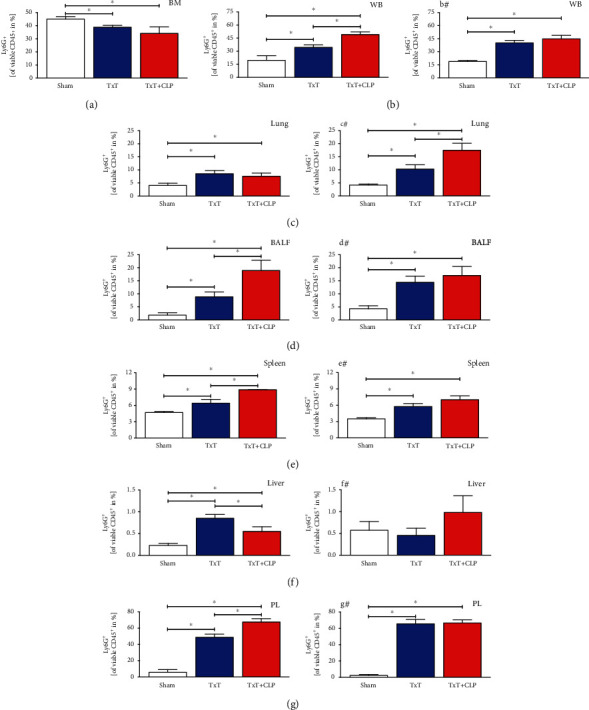
Percental ratio of PMNL fraction among leukocytes in sham (white bars), isolated chest trauma (TxT, blue bars), and systemic inflammation model (TxT + CLP, red bars) was assessed (a) in the bone marrow, (b) in the whole blood (WB), (c) in the lungs, (d) in the bronchoalveolar lavage fluid (BALF), (e) in the spleen (f), in the liver (g), and in peritoneal lavage (PL) by the expression of Ly6G^+^ in flow cytometry. ^∗^*p* < 0.05*vs.* indicated. (b#–g#) Corresponding groups with intratracheal application of the neutralizing CC16 antibody. Data are represented as the mean ± standard error of the mean.

Following blunt chest trauma, the PMNL fraction was significantly reduced in the bone marrow (*p* < 0.05, Figure 3(a)) and significantly increased in the blood, spleen, BALF, lung, PL, and liver (*p* < 0.05, Figures 3(b)–3(g)). After induction of systemic inflammation, the PMNL fraction trended to a further attenuation in the bone marrow but was significantly increased compared to monotrauma in the blood, spleen, BALF, and PL. In the lungs (Figure 3(c)), systemic inflammation resulted in PMNL reduction compared to isolated chest trauma, while in the liver (Figure 3(f)) additional CLP in the TxT + CLP group induced a significant PMNL reduction (*p* < 0.05).

#### 3.1.2. Production and Systemic Release of Immature PMNL Were Induced by Trauma

**Figure 4:**
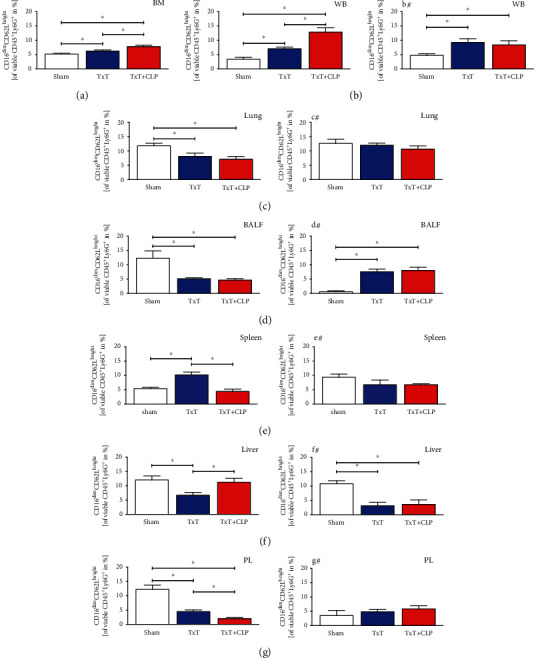
Percental ratio of immature PMNL among the CD11b^+^ PMNL fraction in sham (white bars), isolated chest trauma (TxT, blue bars), and systemic inflammation model (TxT + CLP, red bars) was assessed (a) in the bone marrow, (b) in the whole blood (WB), (c) in the lungs, (d) in the bronchoalveolar lavage fluid (BALF), (e) in the spleen, (f) in the liver, and (g) in the peritoneal lavage (PL) by flow cytometry. ^∗^*p* < 0.05*vs.* indicated. (b#–g#) Corresponding groups with intratracheal application of the neutralizing CC16 antibody. Data are represented as the mean ± standard error of the mean.

In mice undergoing blunt chest trauma, relative levels of immature PMNL (Figure 4) were significantly increased in the bone marrow, blood, and spleen, while systemic inflammation caused further significant enhancement in the bone marrow and blood, but a decrease to sham levels in the spleen (*p* < 0.05, Figures 4(a), 4(b), and 4(e)). TxT induced a significant reduction of immature PMNL in the BALF, lung, PL, and liver (*p* < 0.05, Figures 4(c), 4(d), 4(f), and 4(g)). Following CLP in the TxT + CLP group, levels in the BALF and lung remained decreased, while the level in PL was significantly further attenuated. CLP enhanced the hepatic ratio of immature PMNL to levels comparable to sham (Figure 4(f)).

#### 3.1.3. Circulating Classical PMNL Migrated in Tissues after Isolated Chest Trauma and Systemic Inflammation

**Figure 5:**
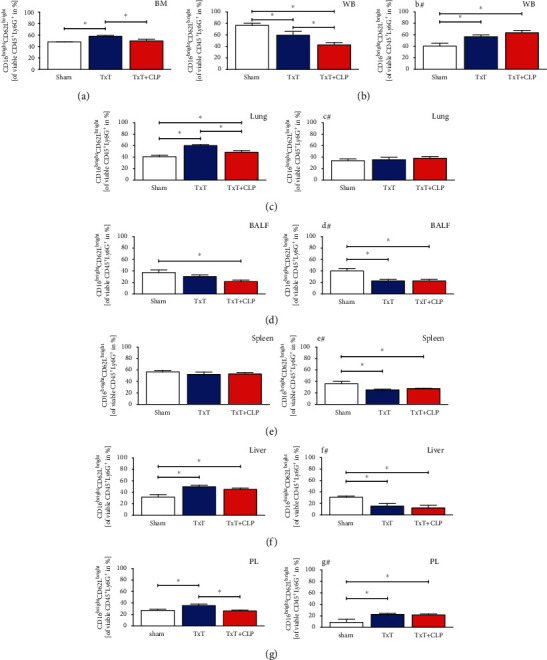
Percental ratio of classical PMNL among the CD11b^+^ PMNL fraction in sham (white bars), isolated chest trauma (TxT, blue bars), and systemic inflammation model (TxT + CLP, red bars) was assessed (a) in the bone marrow (BM), (b) in the whole blood (WB), (c) in the lungs, (d) in the bronchoalveolar lavage fluid (BALF), (e) in the spleen, (f) in the liver, and (g) in the peritoneal lavage (PL) by flow cytometry. ^∗^*p* < 0.05*vs.* indicated. (b#, c#) Corresponding groups with intratracheal application of the neutralizing CC16 antibody. Data are represented as the mean ± standard error of the mean.

Monotrauma induced a significant increase of classical PMNL in the bone marrow, lungs, PL, and liver (*p* < 0.05, Figures 5(a), 5(c), 5(f), and 5(g)) that changed to pretraumatic levels in PL and bone marrow after CLP in the TxT + CLP group. Lung levels (Figure 5(c)) significantly decreased during systemic inflammation but remained significantly elevated compared to sham animals (*p* < 0.05). A similar trend was observed in the liver (Figure 5(f)). Monotrauma and systemic inflammation caused a significant decrease of circulating classical PMNL in the blood and after CLP in the TxT + CLP group in the BALF (*p* < 0.05, Figures 5(b) and 5(d)).

#### 3.1.4. Local Injury Caused Attenuation of Immunosuppressive PMNL in Damaged Tissue and Their Increase in Unaffected Tissue

**Figure 6:**
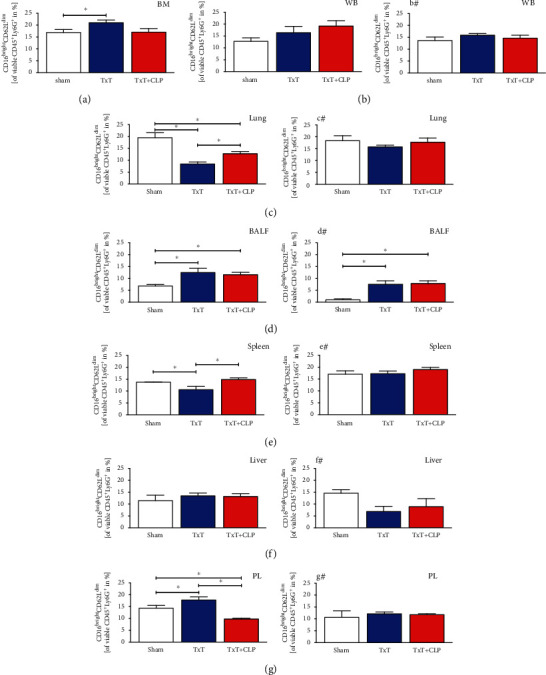
Percental ratio of immunosuppressive PMNL among the CD11b^+^ PMNL fraction in sham (white bars), isolated chest trauma (TxT, blue bars), and systemic inflammation model (TxT + CLP, red bars) was assessed (a) in the bone marrow (BM), (b) in the whole blood (WB), (c) in the lungs, (d) in the bronchoalveolar lavage fluid (BALF), (e) in the spleen, (f) in the liver, and (g) in the peritoneal lavage (PL) by flow cytometry. ^∗^*p* < 0.05*vs.* indicated. (b#–g#) Corresponding groups with intratracheal application of the neutralizing CC16 antibody. Data are represented as the mean ± standard error of the mean.

Following chest trauma, the relative amount of immunosuppressive PMNL (Figure 6) was significantly reduced in the lungs and spleen (*p* < 0.05, Figures 6(c) and 6(e)). Trends to increased levels were detected in the blood and liver (Figures 6(b) and 6(f)), while this effect was significant in the BALF, PL, and bone marrow (*p* < 0.05, Figures 6(a), 6(d), and 6(g)). During CLP-induced systemic inflammation in the lungs in the TxT + CLP group, the ratio was significantly increased compared to isolated chest trauma but remained significantly reduced compared to sham animals (*p* < 0.05, Figure 6(c)). In the blood, there was a further trend to an increase compared to monotrauma and sham (Figure 6(b)). Additional CLP in the TxT + CLP group reduced immunosuppressive PMNL significantly in PL compared to the sham and TxT groups (*p* < 0.05, Figure 6(g)), while the ratio in the spleen and bone marrow normalized towards sham levels (Figures 6(a) and 6(e)).

#### 3.1.5. Local Inhibition of CC16 Antagonizes Posttraumatic Attenuation of Immunosuppressive PMNL

In mice receiving posttraumatic local application of CC16 Ab, distribution of the PMNL fraction increased in the BALF and lungs compared to mice receiving IgG-control antibodies (Figures 3(c), 3(c#), 3(d), and 3(d#)). Pulmonal CC16 neutralization following TxT increased the induction of the PMNL fraction in PL (Figures 3(g) and 3(g#)). Regarding the changing distribution of immature PMNL, the inhibition of CC16 was able to reduce immature PMNL in sham BALF and PL, in the spleen after TxT and in the blood and liver following CLP in the TxT + CLP group (Figures 4(b), 4(d)–4(g), and #, respectively). In mice receiving CC16 Ab compared to mice receiving control antibodies, increases of immature PMNL were observed after CC16 neutralization in BALF. In the lungs (Figures 4(c) and 4(c#)), they increased towards sham levels following TxT and CLP in the TxT + CLP group. Among the ratio of classical PMNL in mice receiving CC16 neutralization, cells were reduced in the spleen, PL, and blood of sham animals (Figures 5(b), 5(e), 5(g), and #, respectively). CC16 neutralization caused an increase of classical PMNL in the blood following CLP in the TxT + CLP group. In the lungs (Figures 5(c) and 5(c#)), the ratio of classical PMNL was not changed through CC16 Ab application after TxT and CLP in the TxT + CLP group. In the liver (Figures 5(f) and 5(f#)), classical PMNL were reduced after TxT and CLP in the TxT + CLP group following CC16 neutralization. Concerning immunosuppressive PMNL, neutralization of CC16 reduced the ratio in sham BALF (Figures 6(d) and 6(d#)), after TxT in the liver and PL (Figures 6(f), 6(f#), 6(g), and 6(g#)), following CLP in the TxT + CLP group in the blood (Figures 6(b) and 6(b#)). CC16 Ab-related increase of immunosuppressive PMNL was only seen in the spleen and lungs after TxT towards sham levels (Figures 6(c), 6(c#), 6(e), and 6(e#)).

#### 3.1.6. Trauma Causes Apoptosis in Damaged and Remote Organs

**Figure 7:**
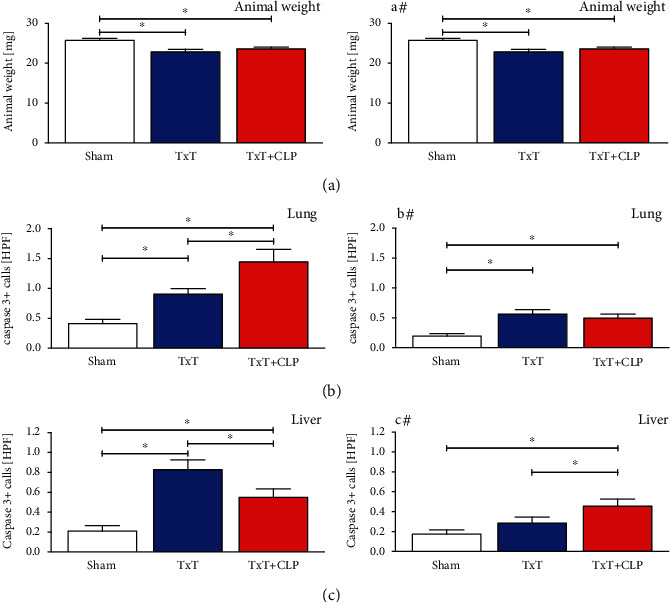
(a) Animal weights in mg of sham (white bars), isolated chest trauma (TxT, blue bars) and systemic inflammation model (TxT + CLP, red bars). (b) Number of apoptotic (caspase-3-positive) cells per high power field (HPF) in lungs. (c) Number of apoptotic cells per high power field (HPF) in liver. (a#–c#): corresponding groups with intratracheal application of the neutralizing CC16 antibody. Data are represented as mean ± standard error of the mean. ∗: p < 0.05 *vs.* indicated.

Apoptotic caspase-3 positive cells were significantly induced by blunt chest trauma in the lungs and liver (*p* < 0.05, Figures 7(b) and 7(c)). In the lung, CLP in the TxT + CLP group significantly increased apoptosis. Apoptosis rates in liver cells were significantly attenuated but remained significantly increased towards sham animals (*p* < 0.05). In comparison to that, the numbers of caspase-3-positive cells in mice receiving pulmonal CC16 neutralization were reduced in the lungs and liver following TxT and in lungs after additional CLP in the TxT + CLP group (Figures 7(b#) and 7(c#)).

## 4. Discussion

### 4.1. Posttraumatic Neutrophil Subset Distribution

Trauma significantly affects the immune system by initiating an early proinflammatory systemic reaction that appears within the first days after trauma. This response to traumatic injury is associated with a strong inflammation that may lead to increased damage of local tissues and remote organ damage [5, 31, 32]. In parallel, an immunosuppressive response is induced, which in fact again promotes PICS and posttraumatic infections [17]. Although both were traditionally described as occurring in sequelae, more recent findings suggest the simultaneous appearance of a proinflammatory and an immunosuppressive reaction [33], thus emphasizing the importance of their early regulation to prevent potential dysregulations and clinical complications. PMNL with their capability to regulate early inflammation are among the first nonresidual cells infiltrating the site of tissue damage and infections. As described before [32], we observed that local blunt chest trauma induces the systemic recruitment of PMNL out of the bone marrow; however, the posttraumatic increase of PMNL in damaged or remote organs [32] must be carefully reconsidered. Considering the systemic PMNL recruitment, potential local dysbalances in the distribution of three heterogeneous PMNL subsets [34] might promote posttraumatic complications. Immature (CD16^dim^CD62L^bright^) PMNL are endotoxin-induced and display a banded nuclear morphology [24]. Compared to other PMNL subsets, they possess the highest intracellular bacterial containment capacity [35, 36]. The largest population among circulating PMNL is classical (CD16^bright^CD62L^bright^) PMNL [24]. These cells are characterized by a segmented nuclear morphology and referred to as mature PMNL [37]. The most recently described immunosuppressive subpopulation (CD16^bright^CD62L^dim^), with a hyper-segmented nuclear morphology, has lower intracellular bacterial containment capacity [36], but is able to suppress the proliferation of immune-meditating T-cells by direct cell-cell interaction [24]. A possible stimulator of human, endotoxin-induced immunosuppressive PMNL is interferon-gamma (IFN-*γ*), as it stimulates the expression of programmed death ligand (PD-L)1. PD-L1 is required in immunosuppressive PMNL to suppress T-cell proliferation via direct cell-cell-interaction [38]. In human and mice, a PMNL subpopulation with upregulated PD-L1 during inflammation provided immunosuppressive characteristics by inducing lymphocyte apoptosis. Interestingly, in the murine PD-L1^high^ population, CD16 was upregulated, while CD62L was downregulated [39], confirming a coexpression of CD16^bright^CD62L^dim^ to indicate immunosuppressive features in human and mice.

Comparable to findings in the human population [40], immature PMNL were induced after TxT and DH in the bone marrow, blood, and spleen (Figures 4(a), 4(b), and 4(e)), while trauma affected their reduction in damaged and remote tissue. It remains elusive, whether trauma-induced reduction in affected and unaffected tissue may be caused by the local differentiation before homing or as seen in the early inflammatory state by a time delay compared to classical PMNL between cytokine release and their recruitment. However, given their higher bacterial containment capacity, increased circulating immature PMNL may potentially inhibit systemic bacterial dissemination. The increase of classical PMNL in several organs following TxT and DH may be induced by trauma-associated danger-associated molecular patterns, which are known to prime PMNL to migrate towards the endothelial barrier and promote their proinflammatory characteristics [41, 42], displaying an early alarmed immune state. Reduction in PL after additional CLP could be a first sign of immune dysregulation in systemic hyperinflammation. Interestingly, blunt chest trauma significantly reduced immunosuppressive PMNL in the lungs, the main site of organ damage. Similar attenuation in the spleen may be associated with the intrathoracic location of this organ with a possible mechanical damage by chest trauma. As the spleen stores PMNL [43], a recruitment of immunosuppressive PMNL after trauma may be assumed. Since the attenuation was only observed in intrathoracic damaged organs, phenotypic changes due to cytokine patterns or emigration may be responsible. An increase of immunosuppressive PMNL in unaffected organs, as shown for PL, bone marrow, blood, and liver following chest trauma might act as an endogenous mechanism providing hyperinflammation. Due to reduced migratory capacity of immunosuppressive PMNL, they might migrate less effectively into affected organs and appear in a higher ratio in unaffected tissue as it was hypothesized before [35]. Attenuation in the PL following additional CLP underlines the declining effects of local damage on immunosuppressive PMNL. The increase of immunosuppressive PMNL following DH in the BALF did not correlate with our findings, suggesting a special role of the BALF in pulmonary injury, underlining recent findings about differences in PMNL behavior in BALF and parenchymal lung tissue [44].

Our data supports the existence of a trauma-induced distribution of the heterogeneous PMNL subsets with distinct characteristics, as highlighted above. Accumulation of cells with specific properties, distinguishing between damaged and unaffected tissues, is a potential method to emphasize inflammation and reduce uncontrolled systemic hyperinflammation after trauma.

### 4.2. Influence of CC16 on the Posttraumatic PMNL Subset Distribution

**Figure 8:**
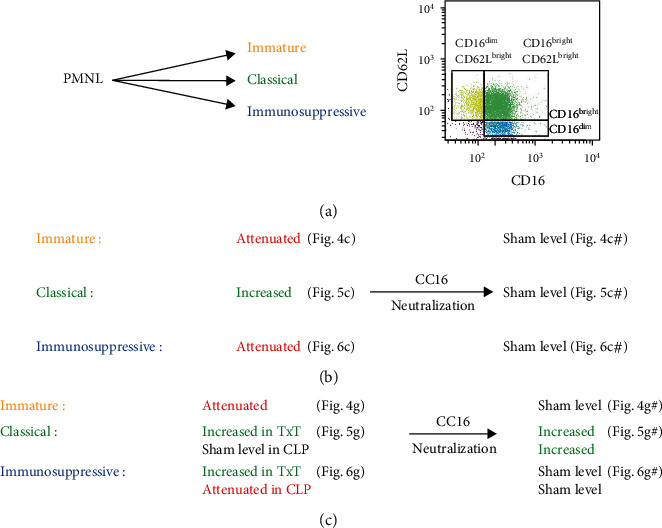
(a) Polymorphonuclear leukocytes (PMNL) were characterized by their relative expression of CD16 and CD62L into the three subsets (immature, CD16^dim^CD62L^bright^; classical, CD16^bright^CD62L^bright^; and immunosuppressive, CD16^bright^CD62L^dim^). (b) Changes in the subset distribution following trauma in the lungs representing affected tissue without and with CC16 neutralization. (c) Corresponding changes in cells in peritoneal (PL) undergoing thoracic trauma (TxT) and additional cecal ligation and puncture (CLP) representing unaffected tissue in TxT and affected tissue in CLP.

More than 50% of severely injured patients with physiologic problems suffer from a severe chest trauma [45], while the last constitutes as a risk factor for complex posttraumatic complications, i.e., acute respiratory distress syndrome [46]. Despite direct damage of the respiratory active tissue, systemic immunomodulation is likely to be involved in trauma-associated late mortality [47, 48]. After local trauma, the systemic effects seem reasonable considering the large contact area between the lungs and blood. CC16 is mainly produced in the lungs [49], and its elevated systemic levels were found in patients with lung damage, correlating with the size of damaged area [8]. Exact functions of CC16 are not entirely understood, but they are closely linked to its anti-inflammatory characteristics, e.g., attenuated IL-8-dependent PMNL chemotaxis [50], inhibited serum phospholipase A2 [51], reduced IFN-*γ* [52], and high affinity to Formyl Peptide Receptor (FPR)2 [53]. As long-term lower serum levels of baseline CC16 in adults are associated with deteriorated lung function [54], little is known about the immunologic role of acutely elevated CC16 levels following chest trauma. The reported correlation of early elevated CC16 levels and elevated CC16 levels in the clinical course with the development of posttraumatic pneumonia [11] suggests that CC16 may act as a promising target to prevent complications in patients suffering from chest trauma. Despite its known immunosuppressive properties, we found that CC16 attenuated immunosuppressive PMNL, as its early neutralization following blunt chest trauma normalized the levels of immunosuppressive PMNL in the lungs. Similar normalizations were found systemically and in the spleen following TxT and in PL after CLP. Corresponding to the elevated rates of immunosuppressive PMNL, rates of classical PMNL were reduced after CC16 neutralization in lungs and spleen. Thus, we suggest CC16 to mediate “proinflammatory” properties during acute lung injury on the PMNL level. This was also highlighted by a reduced number of apoptotic cells after early application of the neutralizing CC16 antibody and supports our recently published data demonstrating that an early CC16 neutralization increased PMNL infiltration but prevented histologic lung damage 24 hours after blunt chest trauma [55]. Early proinflammatory properties are not entirely dissent with the anti-inflammatory properties described above. High local CC16 increase may affect FPR2 interference, as the FPR2 provides proinflammatory properties [56] or the coappearance of proinflammatory cytokines may modulate the immune response towards CC16. Since IFN-*γ* is delayed in its systemic increase compared to other proinflammatory cytokines after trauma [57], CC16 might also provide late immunosuppressive effects by suppressing IFN-*γ*, while the immediate effects on PMNL-differentiation may be rather proinflammatory. Increased rates of immature PMNL following CC16 neutralization in the lungs, BALF, and blood might be associated with reducing effects of CC16 on PMNL migration [58]. On the other hand, it suggests the effect of CC16 to be influenced by the local immune state, as PMNL differentiation differs in unaffected tissue. This is highlighted by the attenuated fractions of immature and classical PMNL in the liver following CC16 neutralization (Figures 4(f) and 4(f#)), associated with a reduced tissue apoptosis in the liver. Taken together, this study is a first approach to characterize the diverse roles of PMNL in inflammation (summarized in Figure 8) and further promising effects of CC16 as a possible factor in their differentiation and as a pharmacologic target in the underlying model. Yet, the exact molecular mechanisms remain elusive.

As limitations of the study, it must be considered that the traditional gating strategy for murine MDSC (Gr-1^+^ and CD11b^+^) with promising human characterization of PMN-MDSC using CD16 and CD62L was applied. Dunay et al. applied CXCR4 and CD62L expression to stratifying two neutrophil populations [59]. Despite findings of murine CD16^bright^CD62L^dim^PD-L1^+^ PMNL with immunosuppressive properties, the comparison of human and murine CD16^bright^CD62L^dim^ PMNL with immunosuppressive characteristics is necessary to allow transferability. One possible issue of identification is the shedding of CD62L [43] as it interferes with CD62L-staining, while shedding could also be a necessary step in the PMNL differentiation. In our gating strategy, we therefore extended the CD62L^dim^ gate towards low, isotype-positive fluorescent intensities, by considering the morphology of relative expression intensity.

## 5. Conclusions

Immature PMNL are released from the bone marrow following trauma and undergo specific subset differentiation at the site of their respective action, differing in damaged and undamaged organs. While classical PMNL were increased in damaged and remote organs, immunosuppressive PMNL were increased in unaffected and attenuated in damaged tissues following local blunt chest trauma (Figure 8(b)). CC16 neutralization affected the differentiation of immunosuppressive PMNL by inhibiting their attenuation in the lungs following TxT. Early CC16 neutralization following blunt chest trauma reduced the amount of apoptotic cells and might therefore be a promising target in preventing the posttraumatic immune dysregulation and improving outcomes.

## Data Availability

Data are available upon a reasonable request to the corresponding author BR.
